# Contribution of antimicrobial stewardship programs to reduction of antimicrobial therapy costs in community hospital with 429 Beds --before-after comparative two-year trial in Japan

**DOI:** 10.1186/2052-3211-7-10

**Published:** 2014-08-05

**Authors:** Tetsuya Fukuda, Hidemi Watanabe, Saeko Ido, Makoto Shiragami

**Affiliations:** Social and Administrative Pharmacy Science, School of Pharmacy, Nihon University, 7-7-1 Narashinodai, Funabashi, 2748555 Japan; Department of Pharmacy, National Hospital Organization, Tochigi Medical Center, 1-10-37 Nakatomatsuri, Utsunomiya, 3208580 Japan

**Keywords:** Antimicrobial stewardship, Community hospital, Cost, Team medicine, Pharmacist

## Abstract

**Objectives:**

Do antimicrobial stewardship programs (ASPs) contribute to reduction of antimicrobial therapy costs in Japanese community hospitals? To answer this health economic question, a before-after comparative two-year trial in a community hospital in the country was designed.

**Methods:**

The study was conducted at National Hospital Organization Tochigi Medical Center, a community hospital with 429 beds. We compared six-month period before-ASP (January 2010 to June 2010) and 24-month period after ASP (July 2010 to June 2012) in primary and secondary outcome measures. Three medical doctors, three pharmacists and two microbiology technologists participate in the ASPs. The team then provided recommendations based on the supplemental elements to primary physicians who prescribed injectable antimicrobials. Prospective audit with intervention and feedback was applied in the core strategy while dose optimization, de-escalation and recommendations for alternate agents and blood cultures were applied in the supplemental elements. The primary outcome was measured by the antimicrobial therapy costs (USD per 1,000 patient-days), while the secondary outcomes included the amount of antimicrobials used (defined daily doses per 1,000 patient-days), sensitivity rates (%) of *Pseudomonas aeruginosa (P. aeruginosa*) to Meropenem (MEPM), Ciprofloxacin (CPFX) and Amikacin (AMK), length of stay (days) and detection rates (per 1,000 patient-day) of methicillin-resistant *Staphylococcus aureus* (MRSA) and extended spectrum beta-lactamase-producing organisms (ESBLs) through blood cultures.

**Results:**

In the study, recommendations were made for 465 cases out of 1,427 cases subject to the core strategy, and recommendations for 251 cases (54.0%) were accepted. After ASP, the antimicrobial therapy costs decreased by 25.8% (*P* = 0.005) from those before ASP. Among the secondary outcomes, significant changes were observed in the amount of aminoglycosides used, which decreased by 80.0% (*P* < 0.001) and the detection rate of MRSA, which decreased by 48.3% (*P* < 0.001).

**Conclusions:**

The study suggested the possibility that ASPs contributed to the reduction of the antimicrobial therapy costs in a community hospital with 429 beds.

## Introduction

The emergence of drug-resistant strains of bacteria has become a global issue, and it is critical to promote appropriate use of antimicrobials in order to prevent the emergence of drug-resistant strains of bacteria. In 2007, Infectious Diseases Society of America and the Society for Healthcare Epidemiology of America published Guidelines for Developing an Institutional Program to Enhance Antimicrobial Stewardship [1]. The Guidelines recommend prospective audit and formulary restriction and preauthorization for core strategy of Antimicrobial Stewardship Programs (ASPs) where dose optimization and de-escalation are listed as supplemental elements for the core strategy. ASPs generally have two goals [1, 2]. One is to optimize clinical outcome while minimizing the emergence of drug-resistant bacteria and incidence of adverse reactions to antimicrobials by promoting appropriate use of antimicrobials, and the other is to reduce healthcare costs. To date, several reports, including studies conducted in Intensive Care Units [3, 4], an World Wide Web-based antimicrobial stewardship program [5] and reports on antimicrobial therapy cost reduction by antimicrobial stewardship teams at university hospitals [6, 7], demonstrated economic effects of ASPs, which are becoming a critical program of team medicine in terms of health economics.

Although the effectiveness of ASPs has been confirmed in many Japanese university hospitals [8, 9], health economic effects of ASPs have not been demonstrated in small-to-medium sized community hospitals, which account for a large proportion of medical institutions in the country.

In other countries, there is a growing recognition of the need for ASPs in community hospitals [10, 11], and it is expected that ASPs will also bring benefits to small-to-medium sized community hospitals in Japan. Do ASPs contribute to reduction of antimicrobial therapy costs in Japanese community hospitals? To answer this health economic question, a before-after comparative two-year trial in a community hospital in the country was designed.

## Methods

### Setting and study design

The study was conducted at National Hospital Organization Tochigi Medical Center, a community hospital with 429 beds. National Hospital Organization Tochigi Medical Center introduced ASPs in July 2010. We compared six-month period before-ASP (January 2010 to June 2010) and 24-month period after ASP (July 2010 to June 2012) in primary and secondary outcome measures.

Medical practice in the study period was carried out under the Japanese National Health Insurance system (NHI). Therefor all medical costs were covered by NHI and patients bear part of the cost based on the NHI rule.

Hospitalized patients subject to ASP were selected from those admitted to High Care Unit and internal medicine, surgery, orthopedics, cerebral surgery, ophthalmology, obstetrics and gynecology, otolaryngology and oral surgery units where pediatric inpatients were excluded.

Three medical doctors (an infection control doctor and two general internists) and three pharmacists, including a trained pharmacist for infectious disease treatment, and two microbiology technologists participate in the ASPs. The team made rounds of patients to ensure appropriate use of antimicrobials for at least once a week.

### Core strategy and supplemental elements of ASPs

**Figure 1:**
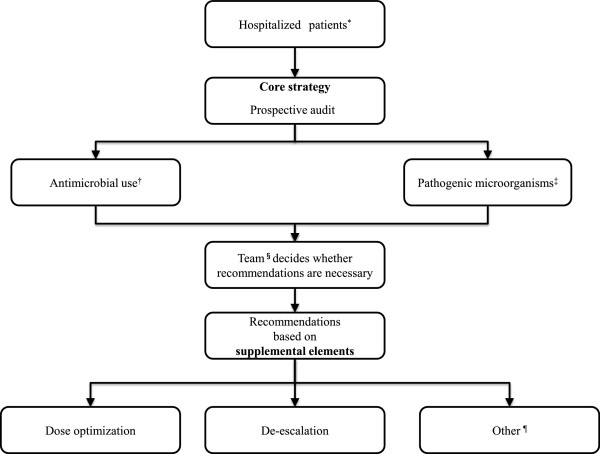
**Antimicrobial stewardship programs flowchart.** ^*^Hospitalized patients: High care, internal medicine, surgery, orthopedics, cerebral surgery, ophthalmology, obstetrics and gynecology, otolaryngology and oral surgery units. ^†^Antimicrobial use: used Glycopeptides or Carbapenems used 3,4-Generation Cephalosporins or Quinolones for 14 days or more. ^‡^Pathogenic microorganisms: Positive blood cultures, Methicillin-resistant *Staphylococcus aureus,* Extended spectrum beta-lactamase-producing organisms, *Acinetobacter baumannii, Pseudomonas aeruginosa* resistant to at least one among Carbapenems, Quinolones or Aminoglycosides ^§^Team: a team comprised of medical doctors, pharmacists and microbiology technologists. The team then provided recommendations based on the supplemental elements to primary physicians who prescribed injectable antimicrobials. Other: recommendations for alternate agents and blood cultres.

Figure 1 describes the flowchart of the ASPs conducted in the study.

In the ASPs conducted in the study, prospective audit with intervention and feedback was applied in the core strategy for hospitalized patients. Patients subject to the core strategy were selected based on the status of antimicrobial use and isolation of pathogenic microorganisms.

According to the status of antimicrobial use, core strategy were performed in patients who used Glycopeptides or Carbapenems or those who used 3,4-Generation Cephalosporins or Quinolones for 14 days or more. According to the status of isolation of pathogenic microorganisms, core strategy were conducted in patients with positive blood cultures, patients in which methicillin-resistant *Staphylococcus aureus* (MRSA), extended spectrum beta-lactamase-producing organisms (ESBLs) or *Acinetobacter baumannii* was detected and patients in which *Pseudomonas aeruginosa* (*P. aeruginosa*) resistant to at least one among Carbapenems, Quinolones or Aminoglycosides was detected.

Dose optimization, de-escalation and recommendations for alternate agents and blood cultures were applied in the supplemental elements for the ASPs. Dose optimization involved drug administration planning based on pharmacokinetics and pharmacodynamics, renal function and drug level monitoring. De-escalation included discontinuation of empiric antimicrobial therapy depending on negative blood culture results. Alternate agents were recommended in consideration of the transition and sensitivity of antimicrobials. Additional blood cultures were recommended to verify sterilization.

### Data collection

The primary outcome was measured by the antimicrobial therapy costs (USD per 1,000 patient-days). Manufacturer list prices were referred to for the antimicrobial drug prices.

The secondary outcomes included the amount of antimicrobials used (defined daily doses per 1,000 patient-days), sensitivity rates (%) of *P. aeruginosa* to Meropenem (MEPM), Ciprofloxacin (CPFX) and Amikacin (AMK) and detection rates (per 1,000 patient-day) of MRSA and ESBLs through blood cultures. Defined daily doses (DDDs) per 1,000 patient-days were calculated to estimate the amount of antimicrobials used. Anatomical Therapeutic Chemical/DDD Index 2012 of the World Health Organization (WHO) Center for Drugs Statistics Methodology was used in the calculation of DDDs. VITEK 2 (Sysmex-bioMérieux Japan, Kobe, Japan) was referred to identify bacterial species and measure drug sensitivity. Minimum inhibitory concentration (MIC) breakpoints were defined in compliance with Clinical Laboratory Standards Institute (CLSI) Standards.

In the study, existing data processed every month by the pharmaceutical department system in the medical center were used to estimate the antimicrobial therapy costs and amount of antimicrobials used. Existing data processed every month by the clinical laboratory department system were used to identify the sensitivity rates of *P. aeruginosa* to MEPM, CPFX and AMK and the detection rates of MRSA and ESBLs. Existing data processed every month by the accounting system were used to identify the number of hospitalized patients, patient-days and length of stay. Patients cannot be identified through these data.

### Statistical analysis

The differences of median values before ASP and after ASP in the antimicrobial therapy costs, amount of antimicrobials used, sensitivity rates of *P. aeruginosa* to MEPM, CPFX and AMK, length of stay and detection rates of MRSA and ESBLs were assessed by the nonparametric Mann–Whitney U-tests. It was regarded as statistically significant when the *P*-value < 0.05. Two-tailed tests were conducted for all the assessments. PASW Statistics (Ver. 18, SPSS Inc., Chicago, IL, USA) for Windows was used in all the statistic calculations.

### Ethical consideration

This study was approved by the Research Ethics Committee of National Hospital Organization Tochigi Medical Center, Utsunomiya.

## Results

### Patients suitable for ASPs

The number of hospitalized patients and patient-days in the six-month period before-ASP totaled 3,025 and 56,479 respectively. The number of hospitalized patients and patient-days in the 24-month period after-ASP totaled 12,654 and 222,388 respectively.

Table 1 shows 1,427 cases (822 males and 605 females, average age of 78.3 years) who underwent core strategy in the 24-month period after-ASP. Among them, 573 cases were selected based on the status of antimicrobial drug use. Of them, 288 cases used Carbapenems, which was most commonly administered among all the antimicrobial drugs used in the study. The remaining 854 cases were selected based on the status of isolation of pathogenic microorganisms. Of them, 360 cases were blood culture positive, which was most commonly observed in the 854 cases.

**Table 1:** **Characteristics of core strategy**

Category	n = 1427
**Status of antimicrobial use n (%)**	**573 (40.2)**
• Carbapenems use	288 (20.2)
• 3,4-Generation Cephalosporins, Quinolones	149 (10.4)
>14 inpatient days	
• Glycopeptides use	136 (9.5)
**Isolation of pathogenic microorganisms n (%)**	**854 (59.8)**
• Blood culture - positive	360 (25.2)
• MRSA	334 (23.4)
• ESBLs	73 (5.1)
• *P. aeruginosa*	71 (5.0)
• *Acinetobacter baumannii*	16 (1.1)

MRSA: methicillin-resistant *Staphylococcus aureus.*

ESBLs: extended spectrum beta-lactamase-producing organisms.

*P. aeruginosa*: *Pseudomonas aeruginosa* resistant to at least one among Carbapenems, Quinolones and Aminoglycosides.

### Recommendations

**Figure 2:**
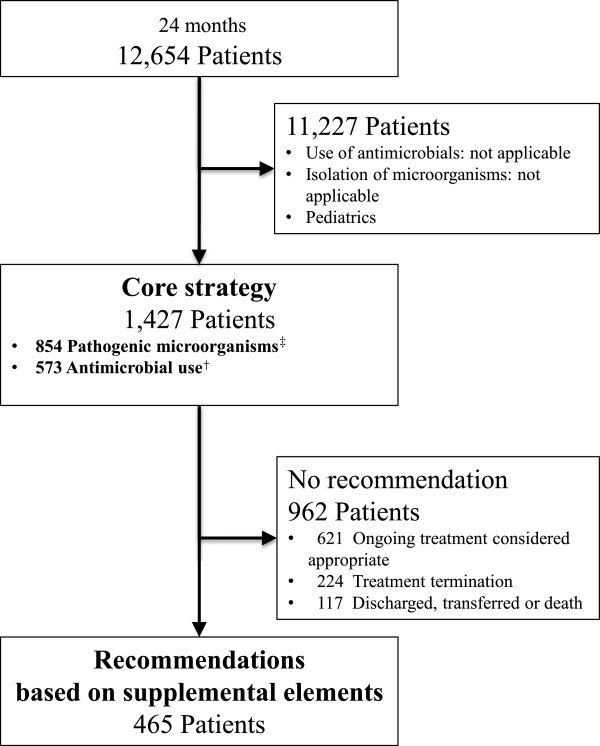
**Recommendation processs flowchart.** ^†^Antimicrobial use: used Glycopeptides or Carbapenems used 3,4-Generation Cephalosporins or Quinolones for 14 days or more ^‡^Pathogenic microorganisms: Positive blood cultures, Methicillin-resistant *Staphylococcus aureus* Extended spectrum beta-lactamase-producing organisms, *Acinetobacter baumannii Pseudomonas aeruginosa* resistant to at least one among Carbapenems, Quinolones or Aminoglycosides.

Figure 2 describes the flow of the recommendation process. Recommendations were provided based on the supplemental elements to 465 cases out of 1,427 cases subject to the core strategy in the 24-month period after-ASP.

Table 2 describes the details of the recommendations provided. Recommendations were accepted for 251 cases (54.0%). Dose optimization was accepted for 110 cases out of 162 cases recommended (67.9%) and de-escalation was accepted for 81 cases out of 208 cases recommended (38.9%).

**Table 2:** **Recommendations based on supplemental elements**

Category	Recommendations	Accepted	Accepted rate (%)
**Total**	465	251	54.0
**Optimize dose**	162	110	67.9
• PK/PD based dose	80	38	47.5
• Renal dose adjustments	67	60	89.6
• Drug level monitoring	15	12	80.0
**De-escalation**	208	81	38.9
**Other**	95	60	63.2
• Blood cultures	74	44	59.5
• Alternate agents	21	16	76.2

PK/PD: Pharmacokinetic and Pharmacodynamics.

Drug level monitoring: Vancomycin and Aminoglycosides were subject to monitoring.

Blood cultures: Additional blood cultures were recommended verify sterilization.

### Primary outcome

**Figure 3:**
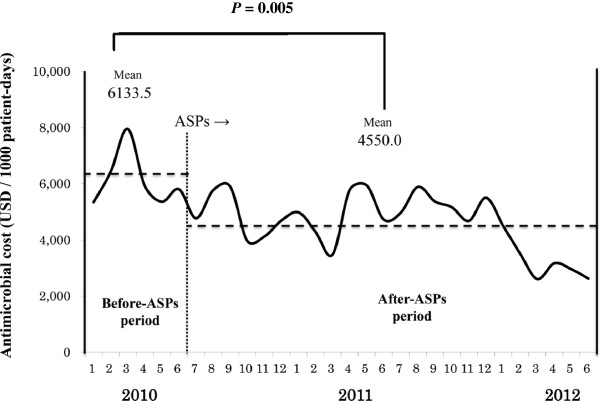
**Antimicrobial cost.** ASPs: antimicrobial stewardship programs. *P* values are used for comparisons of After with Before by Mann–Whitney *U*-test.

Figure 3 shows the antimicrobial therapy costs during the study period. The costs decreased by 25.8% after ASP compared to those before ASP. The mean monthly costs significantly decreased to 4,555.0 from 6,133.5 USD per 1,000 patient-days (*P* = 0.005) (1USD = 98.265JPY).

### Secondary outcomes

Table 3 indicates the amount of antimicrobials used. The mean monthly amount of Aminoglycosides used significantly decreased to 0.6 DDDs per 1,000 patient-days after ASP from 3.0 before ASP (*P* < 0.001). The mean monthly amount of Quinolones used decreased to 2.0 DDDs per 1,000 patient-days after ASP from 4.0 before ASP (*P* = 0.09). The amount of Penicillins used increased to 35.3 DDDs per 1,000 patient-days after ASP from 22.7 before ASP (*P* = 0.06).

**Table 3:** **Antimicrobial use**

	Mean		DDDs per 1000 patient-days
	**Before**	**After**	***P*** **value**
**Total**	138.7	138.8	0.96
**Aminoglycosides**	3.0	0.6	< 0.001
**Penicillins**	22.7	35.3	0.06
**Quinolones**	4.0	2.0	0.09
**1,2-Generation Cephalosporins**	54.6	53.2	0.44
**3,4-Generation Cephalosporins**	33.1	29.0	0.16
**Carbapenems**	17.5	15.8	0.21
**Glycopeptides**	3.1	2.4	0.16
**Antifungals**	0.7	0.5	0.35

DDDs: Defined Daily Doses (WHO Center for Drug Statistics Methodology).

*P* values are used for comparisons of After with Before by Mann–Whitney *U*-test.

The mean monthly sensitivity rate of *P. aeruginosa* to MEPM changed to 88.5% after ASP from 84.0% before ASP (*P* = 0.21) while that to CPFX changed to 75.3% from 74.8% (*P* = 1.00) and that to AMK changed to 99% from 100% (*P* = 0.37).

The mean monthly length of stay declined to 15.9 days after ASP from 16.6 days before ASP (*P* = 0.09). The monthly detection rate of MRSA significantly decreased from 2.9 to 1.5 per 1,000 patient-days (*P* < 0.001) while that of ESBLs decreased from 0.4 to 0.3 per 1,000 patient-days (*P* = 0.38).

## Discussion

The study revealed two findings. Firstly, ASPs contributed to the reduction of antimicrobial therapy costs in the Japanese community hospital with 429 beds. Secondly, ASPs possibly helped prevent reduction in the sensitivity rates of *P. aeruginosa* to MEPM, CPFX and AMK and contributed to decreasing the detection rate of MRSA.

The antimicrobial therapy costs decreased after ASP, which indicated the significance of ASPs in the community hospitals with 429 beds. In response to the recommendations, including de-escalation and dose optimization, which were provided for the cases selected through the prospective audits, the use of less expensive Penicillins was on the increase while the use of other antimicrobials decreased or was on the decrease. As a result, the antimicrobial therapy costs significantly decreased after ASP compared to those before ASP. In another study conducted in a community hospital with 535 beds, Malani et al. reported a 13.3% reduction of antimicrobial therapy costs in cost-per patient-days through prospective audits of some antimicrobial drugs for 12 months [12]. Storey et al. also reported a 25% reduction of antimicrobial therapy costs in cost per patient-days through 16-month ASP in a community hospital with 100 beds [13]. In this study conducted at Tochigi Medical Center, the antimicrobial therapy costs decreased by 25.8%. The length of the investigation period, which extended for two years, added credibility to the study.

In the study, the amount of Aminoglycosides used significantly decreased and that of Quinolones was on the decrease after ASP. Defez et al. suggested that an odds ratio of 4.7 in the history of Quinolones use until seven days before can be a risk factor for hospital-acquired infections with multidrug-resistant *Pseudomonas aeruginosa* in *P. aeruginosa* infected patients [14]. In the light of the risk factor reported by Defez et al., the results of the study were considered to be preferable. In addition, it was suggested that the study possibly prevented reduction in the sensitivity rates of *P. aeruginosa* to MEPM, CPFX and AMK. The study results also supported the report made by Dunbar LM et al., which indicated the possiblity that dose optimization contributed to decreasing resistant bacteria [15]. The study results were considerd to be clinicially significant as Carmeli Y et al. reported increases in the mortality rate and length of stay associated with the emergence of resistant *P. aeruginosa* [16]. As the length of stay was on the decrease after ASP in the study, ASPs were expected to bring about economic effects while a prospective payment system on a fixed fee basis is being introduced more widely in inpatient care. Furthermore, Fraser GL et al. reported a decrease in the length of stay from 24.7 days to 20 days due to recommendations given by infectious disease specialists and pharmacists for optimal use of antimicrobials [17]. In the study, it was considered that the team rounds to promote appropriate use of antimicrobials contributed to enhancing the compliance with standard prevention measures among healthcare professionals, which ultimately led to the reduction in the detection rate of MRSA through blood cultures.

In addition to ASPs, the Antimicrobial Stewardship Guidelines provided by the Infectious Diseases Society of America and the Society for Healthcare Epidemiology of America [1] strongly recommend formulary restriction and preauthorization as well as conversion of antimicrobials from parental to oral. Furthermore, Bauer KA et al. reported a 1.7-day reduction in the length of stay in patients with *Staphylococcus aureus* bacteremia through rapid diagnosis using rapid PCR testing [18]. Yam P et al. also reported a decreae of *Clostridium difficile* infections from 5.5 cases per 10,000 patient-days to 1.6 cases through the application of telemedicine technology in a rural hospital [19]. By combining these initiatives with ASPs, more effective prevention of resistant bacteria and greater economic effects can be expected.

This study has limitations in three aspects. Firstly, only one facility was considered in the study. As clinical knowledge varies by individual medical doctors and pharmacists and isolation frequency of bacteria and drug sensitivity vary among facilities, the study involves selection bias. Secondly, costs other than the antimicrobial therapy costs were not examined in the study. By inhibiting development of *P. aeruginosa* resistance, additional treatment costs were possibly contained. However, the association with cost containment cannot be defined as costs other than the antimicrobial therapy costs were not investigated. Lastly, the relation between ASPs and primary diagnosis or co-morbidities was not considered.

## Conclusion

The study suggested the possibility that ASPs contributed to the reduction of the antimicrobial therapy costs in the community hospital with 429 beds. It is likely that more effective prevention of resistant bacteria and greater economic effects can be achieved through a combination of other ASPs. It is considered worthwhile to study patient prognosis and changes in the incidence of adverse reactions with enhanced ASPs.
